# A novel enzyme-linked immunostaining technique based on silk membrane for the prenatal detection of fetomaternal haemorrhage

**DOI:** 10.3389/fbioe.2023.1175345

**Published:** 2023-05-26

**Authors:** Xinyang Li, Miyang Li, Shengbao Duan, Hongmei Wang, Yong Li, Tiemei Liu

**Affiliations:** ^1^ Department of Blood Transfusion, China-Japan Union Hospital of Jilin University, Changchun, China; ^2^ Department of Laboratory, China-Japan Union Hospital of Jilin University, Changchun, China; ^3^ CAS Key Lab of Bio-Medical Diagnostics, Suzhou Institute of Biomedical Engineering and Technology, Chinese Academy of Sciences, Changchun, China

**Keywords:** silk cocoon membrane, enzyme-linked immunosorbent assay (ELISA), biotin-avidin, fetomaternal haemorrhage (FMH), fetal red blood cells (RBCs)

## Abstract

**Objective:** Developing a simple, rapid, reliable, sensitive, and cost-effective method for prenatal detection of fetomaternal haemorrhage by combining multi-aperture silk membrane with enzyme-linked immunosorbent assay (ELISA), which does not require any complicated instruments and can be visually colored, so as to provide a new method for clinical detection of fetomaternal haemorrhage.

**Methods:** As a carrier, a chemically treated silk membrane was used to immobilize anti-A/anti-B antibody reagent. PBS washed slowly after vertically dropping red blood cells. After adding biotin-labeled anti-A/anti-B antibody reagent, PBS is slowly washed, enzyme-labeled avidin is added, and TMB is used for color development after washing.

**Results:** When there were both anti-A and anti-B fetal erythrocytes in pregnant women’s peripheral blood, the final color was dark brown. When there are no anti-A and anti-B fetal red blood cells in pregnant women’s peripheral blood, the final color development results do not change, which corresponds to the color of chemically treated silk membrane.

**Conclusion:** The new enzyme-linked immunosorbent assay (ELISA) based on a silk membrane can distinguish fetal red blood cells from maternal red blood cells prenatally and can be used for prenatal detection of fetomaternal haemorrhage.

## 1 Introduction

Two or three out of every 10,000 pregnant women are reported to have FMH (fetomaternal haemorrhage) (Tao et al., 2022). The essence of detecting FMH is the quantitative detection of fetal red blood cells in the peripheral blood of pregnant women (Troia et al., 2019). Prenatal detection of maternal hemorrhage is the foundation for preventing newborn hemolytic disease and administering immunoglobulin therapy (Christino Luiz et al., 2019; Cormack et al., 2019; Flynn et al., 2022).

**FIGURE 1:**
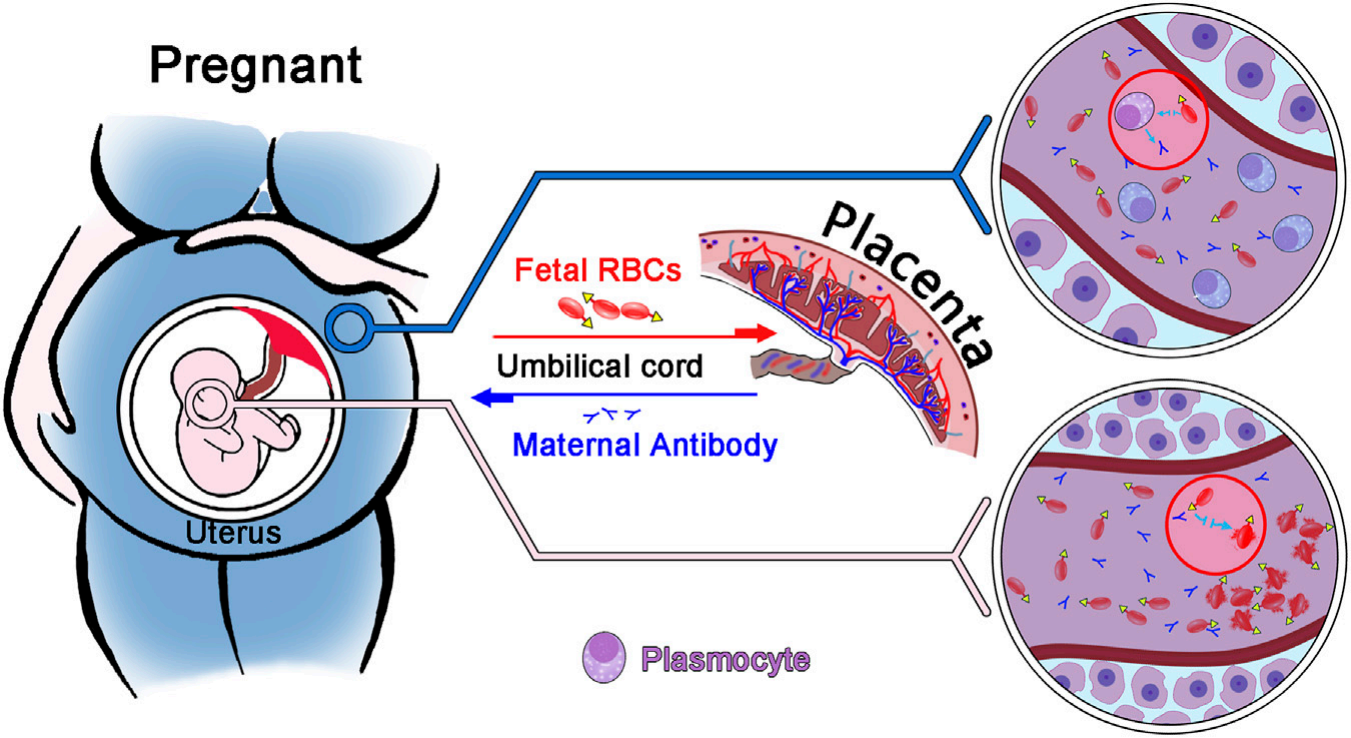
Diagram illustrating the principle of fetomaternal haemorrhage. Through the placenta, fetal red blood cells enter the mother, and the mother produces an IgG antibody against fetal red blood cells. This antibody enters the fetus through the placenta, causing red blood cell hemolysis in the fetus.

FMH refers to the entry of fetal red blood cells into the mother’s body as a result of a compromised placental barrier. Pregnant women produce specific antibodies against incompatible fetal red blood cell antigens, which act on fetal red blood cells and result in their hemolysis (Simoes et al., 2022) (Figure 1). It may lead to fetal anemia, jaundice, edema, and even death (Carr et al., 2022). There are numerous causes of placenta rupture, but the most common are trauma and intrauterine death (Davis, 2019; Hsia et al., 2019; Hookins and Vatsayan, 2020; Monteiro et al., 2021). In the United States, 40% of pregnant women die from trauma, according to statistics (Krywko et al., 2022). In addition, if the fetus survives during pregnancy, mothers with incompatible blood types can produce specific IgG antibodies against fetal red blood cells, which can cause hemolysis in fetal red blood cells *via* the placenta. Nonetheless, if the fetus survives, the newborn may still develop hemolytic disease or pass away after a few months (Akorsu et al., 2019; Song et al., 2021). Common antibodies that can easily cause fetal bleeding include anti-A, anti-B, anti-D, anti-Kell, anti-E, and anti-C antibodies (Pal and Williams, 2015; Erhabor et al., 2020; Minuk et al., 2020; Tneh et al., 2021). 10% of fetal deaths are attributed to maternal bleeding, according to statistical evidence. Therefore, early detection of FMH and treatment with immunoglobulin can reduce fetal mortality (Glazebrook et al., 2020).

Indicators for the detection of FMH consist primarily of fetal red blood cell antigen, fetal hemoglobin, and gene detection (Athiel et al., 2020; Zhang et al., 2021). Fetal red blood cell antibody is utilized to identify fetal red blood cells based on the difference between fetal and maternal blood types. Currently, the most common methods for detecting fetal red blood cells in the mother’s blood are the rosette test (Kim and Makar, 2012) and the microcolumn gel test (Agaylan et al., 2007; Ben-Haroush et al., 2007). Nevertheless, the sensitivity of the rosette test is low, and the microcolumn gel technique cannot be used as a routine clinical detection method due to its complex operation and high material cost. Therefore, it is crucial to establish a method that is sensitive, inexpensive, simple, and quick.

ELISA based on silk membrane developed in this experiment has the following benefits: high sensitivity, low cost, simplicity, rapidity, no instrument required, and visual visualization of chromogenic results. As a natural protein polymer with non-toxicity, non-irritation, biodegradability, biocompatibility, and mechanical properties, natural silk is indispensable not only in the textile industry but also in biomedicine (Wang et al., 2019; Jaleel et al., 2020; Patel et al., 2020; Rodriguez et al., 2020). Silk can be used as a material for sutures, biological scaffolds, and tissue engineering transplants (Feng et al., 2020; Yonesi et al., 2021). In addition to the aforementioned properties, silk is produced by domestic silkworms, making it a readily available and inexpensive material (Ni et al., 2019). Silk is composed of a fibrous protein (fibroin) and a glue protein (sericin) (Song et al., 2020). Silk protein is extracted from the cocoon of the silkworm and is immunogenic (Wang et al., 2021). As a globulin on the surface of silk fibroin, sericin has excellent water-solubility (Wray et al., 2011). Degumming can therefore be used to eliminate sericin, as it does not alter its biocompatibility, immunogenicity, or other properties (Lopez Barreiro et al., 2019).

In this experiment, ELISA developed by silk membrane is simple, rapid, inexpensive, harmless to the health of the fetus and mother, does not require any instruments or equipment, and the results are straightforward to observe. This technique is one type of immunosensor analysis, which combines antigen, antibody, and analyte of interest. In addition to the immune activity and mechanical properties of natural silk, its three-dimensional, multilayered pore structure is also utilized. This structure allows red blood cells with an average diameter of 7.2 microns to pass through without obstruction.

## 2 Materials and methods

### 2.1 Reagents, materials, monoclonal antibodies

The experimental silk film was acquired at the Suzhou market. Anti-silk membrane monoclonal antibody and cross-linked antibody were from the Suzhou Institute of Biomedical Engineering and Technology, Chinese Academy of Sciences. And their preparation methods were consistent with those of Wang’s article. The antibodies’ specificity, affinity, and purity have been confirmed. From Shanghai Hematology, anti-A and anti-B reagents were acquired. Biotin-avidin was purchased from Beijing Biodragon Immunotechnologies Co.,Ltd. The blood sample came from China-Japan Union Hospital of Jilin University and has been approved by the hospital’s Ethics Committee (approval number:20,220,425,011). It is stored at 4°C. Freeze dryer (ALPHA1-4/LD plus) was purchased from Beijing Boli Instrument Co., Ltd.

### 2.2 Study design

**FIGURE 2:**
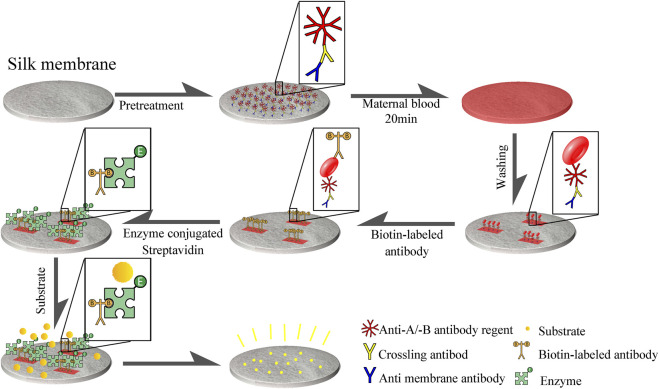
Procedure for detecting FMH using ELISA based a silk membrane. Disk-shaped silk membrane was treated with mouse anti-silk membrane antibody, cross-linked antibody (sheep anti-mouse IgG immunoglobulin), and anti -A/anti -B antibody reagent to form an immune complex structure consisting of anti-silk antibody, cross-linked antibody, and anti -A/anti -B antibody reagent that was connected to silk membrane. Red blood cell antigen that can be coupled with anti-A and anti-B antibodies will be attached to a silk membrane, and then biotin-biotin will be employed.

The natural silk membrane was cut into a disc (with an aperture of 10 mm), soaked in hot water, and the fluffy filaments and the bottom layer of membrane (which is difficult for red blood cells to penetrate) were removed to produce a carrier with the same thickness and aperture. Soak with mouse anti-silk membrane antibody (2H3) followed by cross-linked antibody (sheep anti-mouse IgG immunoglobulin). Specific affinity was then employed to attach the two antibodies to the surface of the silk membrane. Then, anti-A antibody and anti-B antibody were coupled to form a silk membrane capable of recognizing type A red blood cell antigen and type B red blood cell antigen. The non-A-type and non-B-type red blood cells cannot be combined with the chemically treated silk membrane, but they will fall off and separate from the membrane after PBS washing, whereas the red blood cell antigen that has been specifically combined with the silk membrane surface will not fall off. In addition, when precipitated TMB was added to a solution containing biotin-avidin coupled with anti-A/anti-B antibody, distinct hues will be observed. Figure 2 illustrates experimental steps of the silk membrane-based ELISA.

### 2.3 Validation of the multilayer porous properties of silk film

Under an electron microscope, the cross-sections and surfaces of untreated natural silk membrane and chemically treated silk membrane were observed.

### 2.4 Preparation and chemical treatment of silk membrane

The natural silk membrane was formed into a 10-mm-diameter disc using a 10-mm-diameter hole punch, soaked in hot water to remove wax and magazines, and the loose silk fibers were removed by hand. Soak the treated silk film in a chemical treatment solution for 2 h in a water bath at 56°C. Vacuum twice. Soak at 56°C for 2 h. The PBST was cleaned thrice. Soak overnight at 4 °C with 100 g/mL anti-silk membrane antibody. The PBST was cleaned thrice. 5% skim milk powder was sealed for 2 h at 37°C. The PBST was cleaned thrice. The silk membrane was then soaked overnight at 4°C in 200 g/mL of cross-linked antibody. Wash PBS three times, each time gently absorbing water with filter paper, and dry.

### 2.5 Validation of the coupling of the anti-silk membrane antibody and the cross-linked antibody

HRP-labeled sheep anti-mouse IgM and HRP-labeled sheep anti-mouse IgG were incubated at 37°C for 1 h with silk membrane coupled with anti-silk membrane antibody and silk membrane coupled with cross-linked antibody, and then evenly mixed every 15 min. The PBST was washed thoroughly five times. TMB was used to evaluate the coupling of anti-silk membrane antibody and cross-linked antibody.

### 2.6 Validation of the permeability of a chemically treated silk membrane

The red blood cells were vertically dripped onto the chemically treated silk membrane, the red blood cell distribution on the front and back of the membrane was observed, and the membrane’s permeability was compared to commercially available NC membrane and natural untreated silk membrane.

### 2.7 Preparation of biotin-coupled anti-A and anti-B antibodies

Biotin-coupled anti-A and anti-B antibodies were prepared and stored in accordance with the biotin labeling kit’s instructions (Beijing Biodragon Immunotechnologies Co., Ltd.).

### 2.8 Detection of FMH by silk membrane-based ELSA

0.1%, 0.3%, 0.5%, and 1% of fetal red blood cells were mixed with adult red blood cells (the ratio of fetal red blood cells to mixed red blood cells composed of fetal red blood cells and adult red blood cells). 10 L of mixed red blood cells were dripped vertically onto the front of a silk membrane, left to stand for 2 min, and then vertically washed with PBST until the negative control displayed untreated color (positive control only added fetal red blood cells, negative control only added adult red blood cells). Adding biotin-coupled anti-A and anti-B antibodies, washing, adding enzyme-labeled avidin, washing, and then treating in the dark for 2 min with TMB. Observe the outcomes. In this experiment, the biotin-streptomycin-treated silk film was simultaneously analyzed with ImageJ software, and the results were quantified. The data were then plotted using GraphPad Prism 9.

### 2.9 The confirmation of the establishment of a silk membrane-based ELSA for detecting FMH

While detecting fetal red blood cells and adult red blood cells by silk membrane-based ELSA, flow cytometry detected the same portion of fetal red blood cells and adult red blood cells simultaneously. The detection method for FMH using anti-fetal red blood cell antibodies is consistent with that in Kim’s article. Using a parallel control of fetal red blood cells and adult red blood cells by a silk membrane-based ELSA and flow cytometry, the accuracy of negative and positive photographs was confirmed, and the silk membrane-based ELSA was successfully validated to detect FMH.

### 2.10 Sensitivity analysis of a silk membrane-based ELSA and K-B test

In addition, the above method is compared to the traditional K-B test to determine their sensitivity. During the K-B test, 10,000 cells were observed under a microscope and the number of fetal red blood cells was counted in strict accordance with the AABB technical manual.

## 3 Results

### 3.1 Silk membrane structure under electron microscope

**FIGURE 3:**
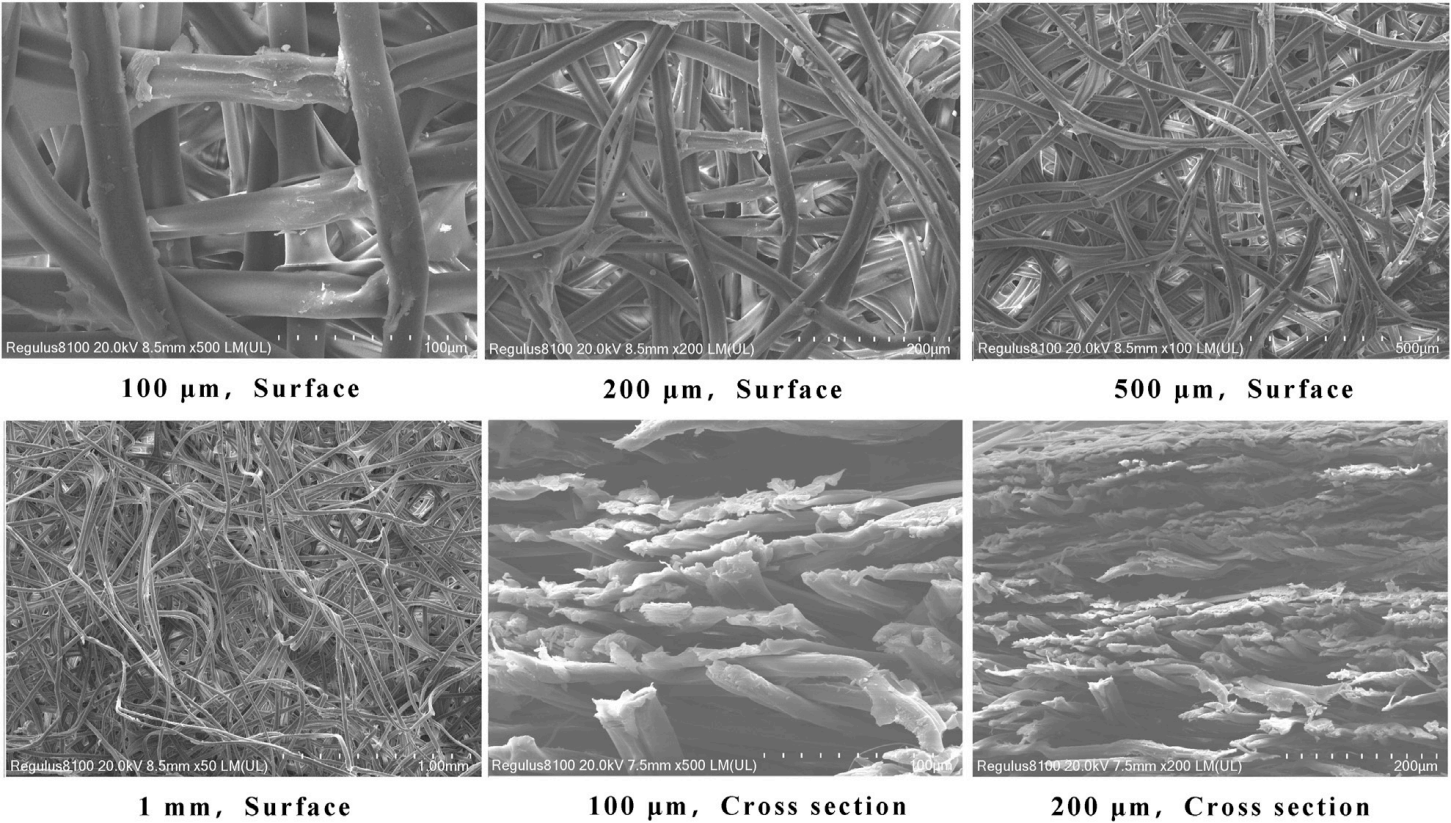
Electron microscopy of the morphology of a silk membrane. 3a∼3 days are top views of silk membrane at various magnifications, whereas 3e and 3f are cross sections of silk membrane at various magnifications.

Under an electron microscope, it is evident that the silk membrane consists of multiple layers of porous membrane that are interwoven with individual fibers. A structure with multiple layers can absorb more carriers and allow red blood cells to permeate the silk membrane. Moreover, compared to a single layer, the multi-layer structure can absorb more red blood cells, increase the adsorption space of antigen-antibody immune complexes, and improve visual results (Figure 3).

### 3.2 Description of the coupling of anti-silk membrane antibody and cross-linked antibody

**FIGURE 4:**
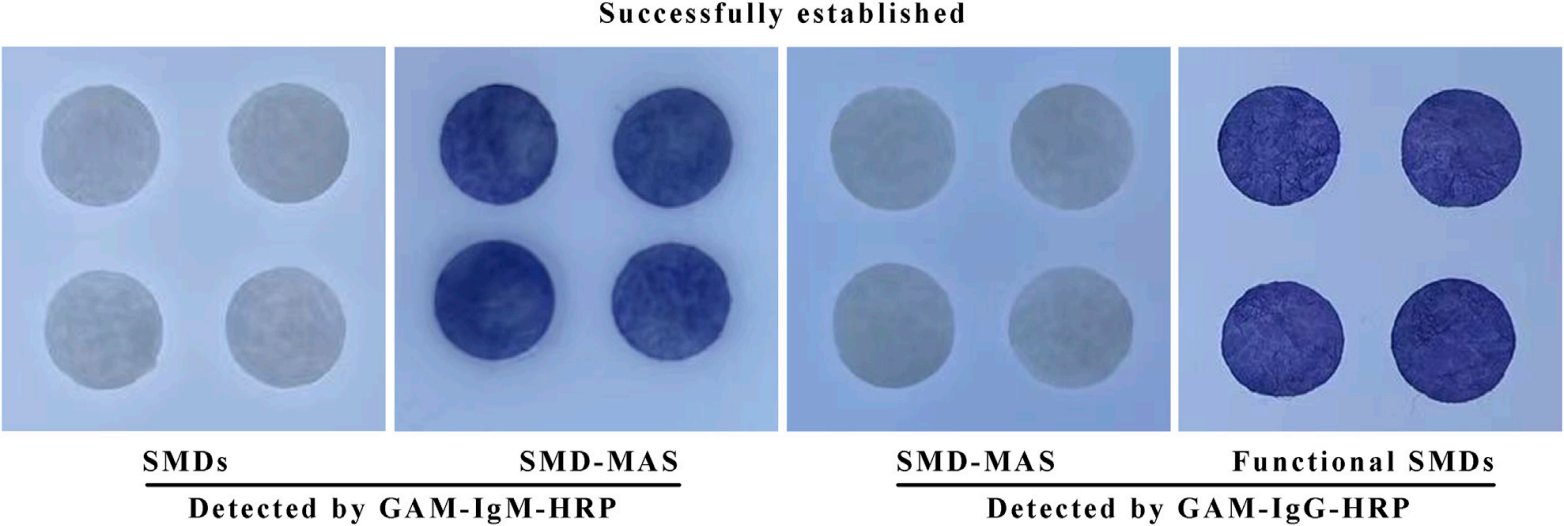
The results of the coupling of anti-silk membrane antibody and cross-linked antibody to silk membrane using GAM—HRP.

Anti-silk membrane antibody and cross-linked antibody are antibodies of the mouse that can react with HRP labeled anti-mouse globulin. After the anti-silk membrane antibody or cross-linked antibody is fixed to the silk membrane, the color of the silk membrane becomes blue after the Goat anti-mouse-globulin and the color developer are added; However, the color of the silk film without anti-silk film antibody or cross-linked antibody was not changed even if Goat anti-mouse-globulin and chromogenic agent were added. Figure 4 shows that the antibodies were successfully coupled to the silk membrane. It indicates that this experimental step has been successfully established.

### 3.3 Permeability of chemically modified silk membrane

**FIGURE 5:**
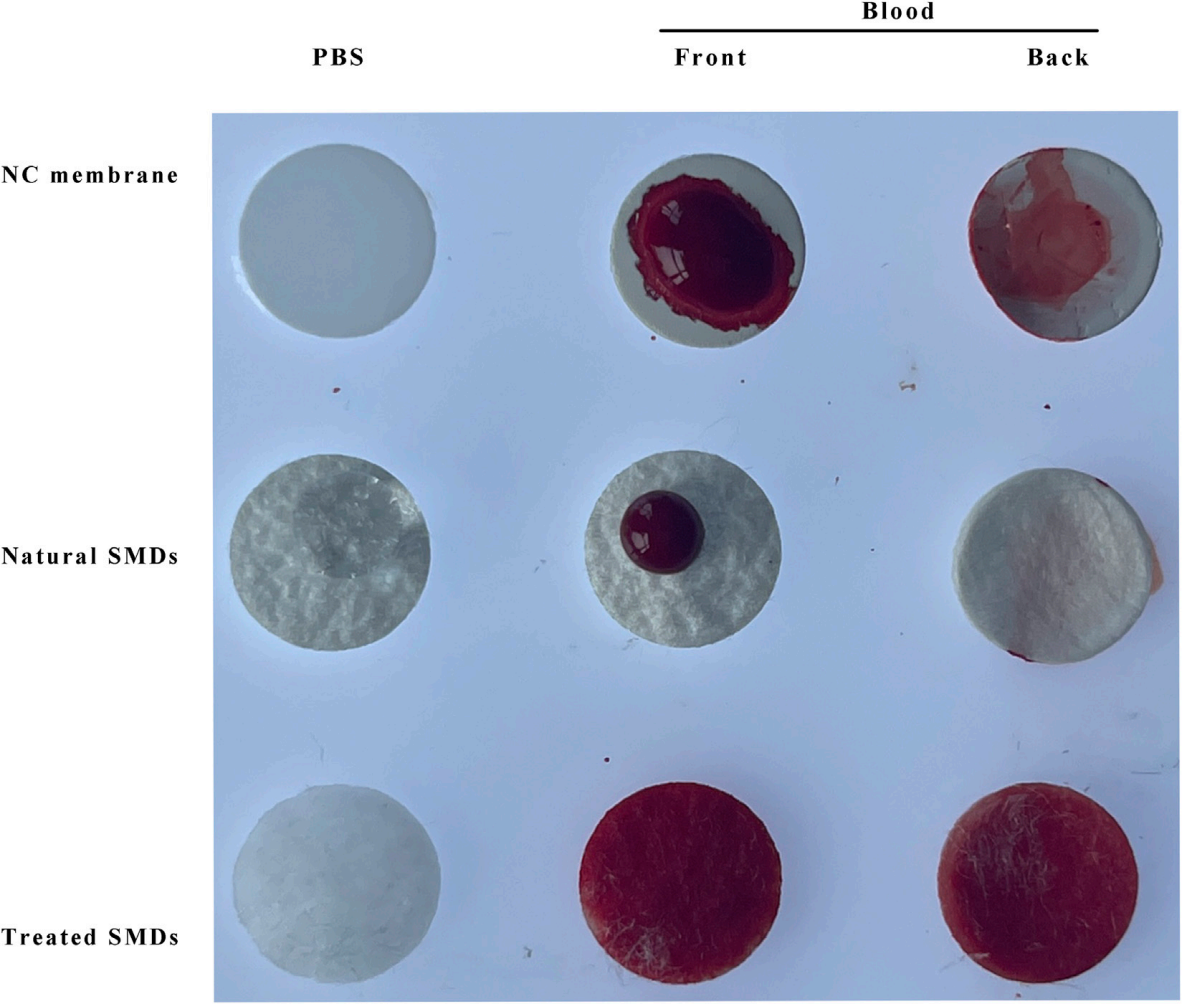
Permeability comparison of NC membrane, natural silk membrane, and chemically treated silk membrane. SMD: silk cocoon membrane disc; MAS: Mouse anti-silk proteins; GAM: goat anti-mouse.

Figure 5 demonstrates that the chemically treated silk membrane can fully permeate both PBS and red blood cells, and that red blood cells can cover both the front and back of the membrane. However, natural silk membrane that has not been treated is impermeable to PBS and red blood cells, whereas NC membrane can pass through PBS but not red blood cells. Therefore, the chemical treatment of silk film in this experiment is beneficial to the experiment’s progression.

### 3.4 Verification of the successful establishment of an ELISA based on silk membrane

**FIGURE 6:**
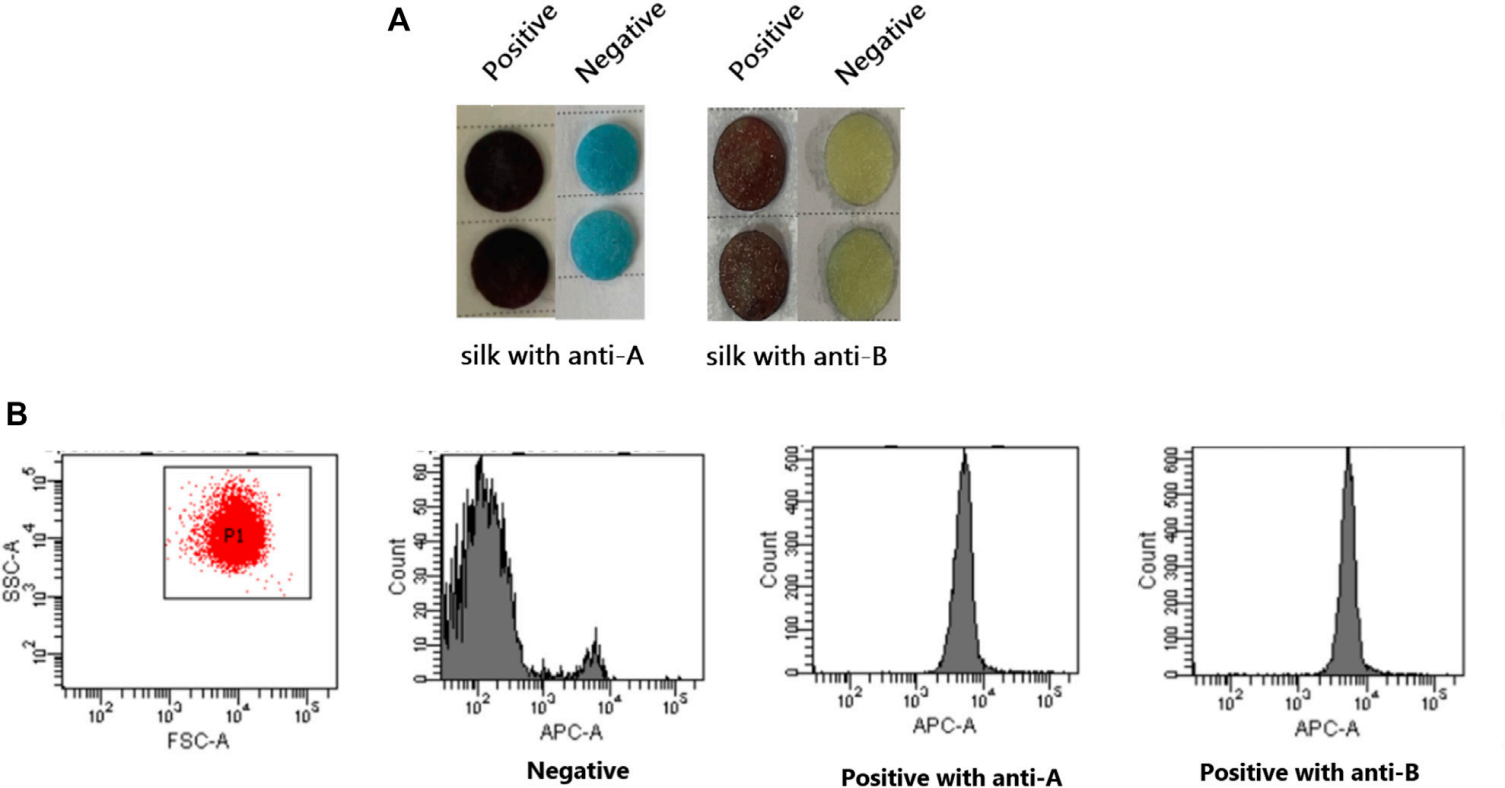
Confirmation of the successful establishment of an ELISA based on a silk membrane to detect FMH by flow cytometry. **(A)**: The negative and positive FMH results detected by ELISA based a silk membrane. **(B)**: The negative and positive FMH results detected by flow cytometry.

Using a novel technique and flow cytometry, identical fetal and adult red blood cells were identified. The silk membrane in Figure 6 was blue and yellow for the negative result and brown for the positive result. At this time, flow cytometry results indicated that the fluorescence intensity of positive results was greater than that of negative results. This demonstrated that the ELISA based on silk membrane has been successfully established and it was reliable and accurate for the detection of FMH.

### 3.5 Detection of various amounts of FMH by ELISA based on silk membrane

**FIGURE 7:**
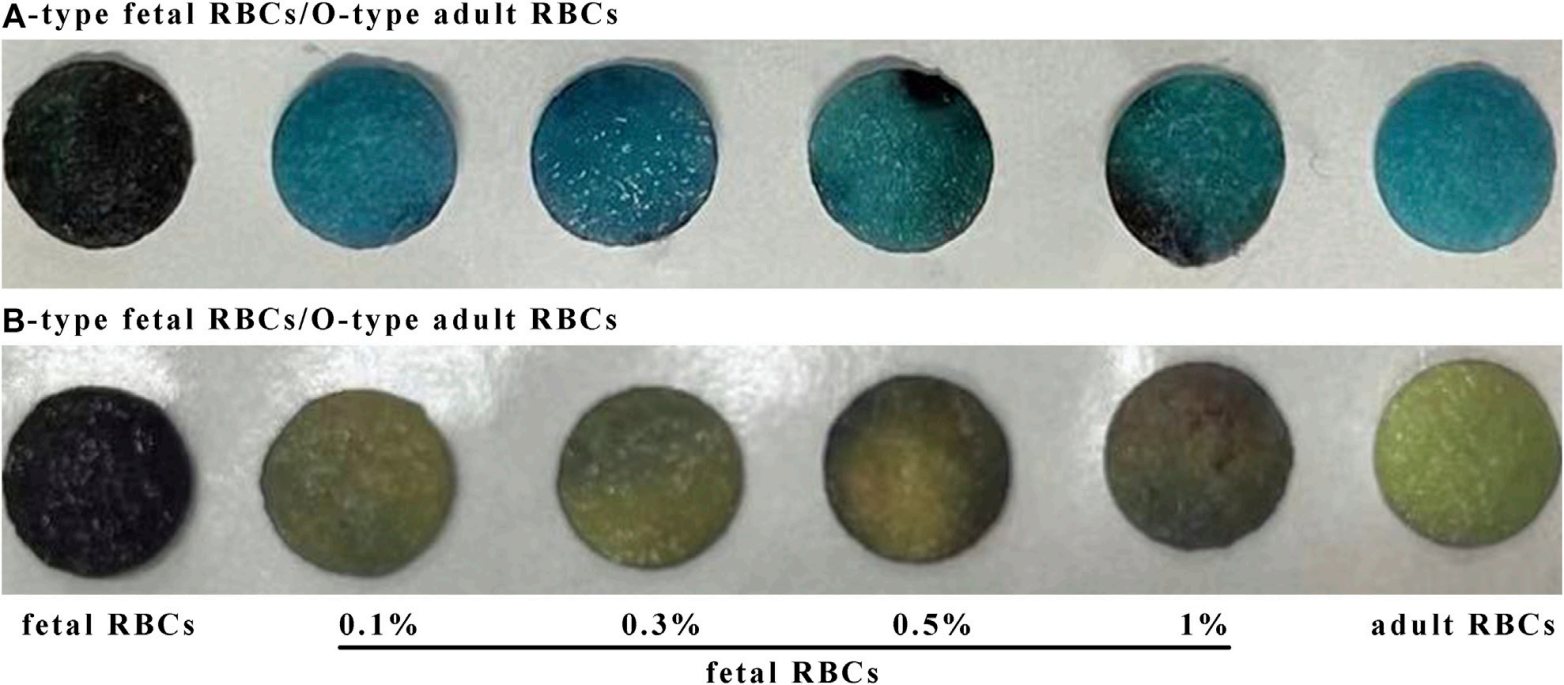
Detection results for various percentages of FMH using an ELISA based on a silk membrane.

**FIGURE 8:**
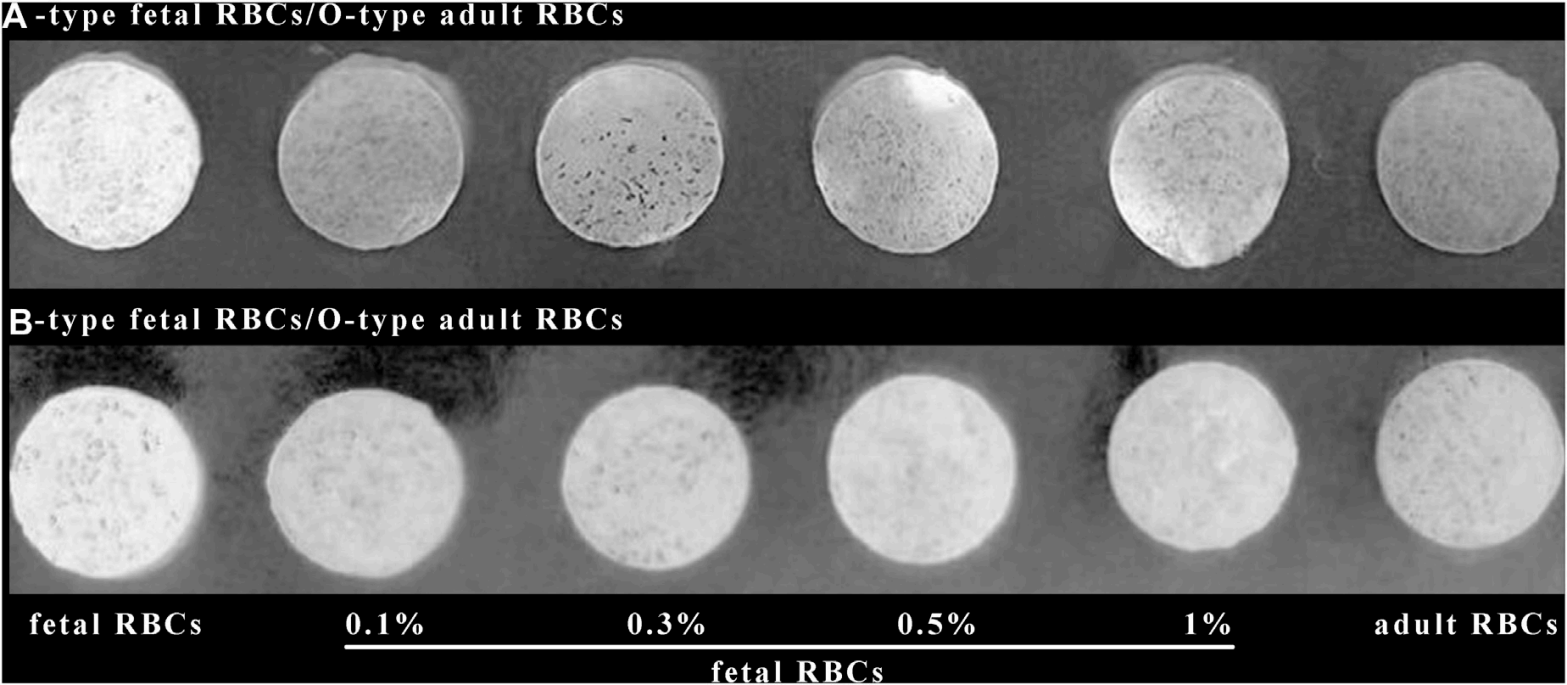
Using ImageJ to blue channel the results of an ELISA based on a silk membrane.

**FIGURE 9:**
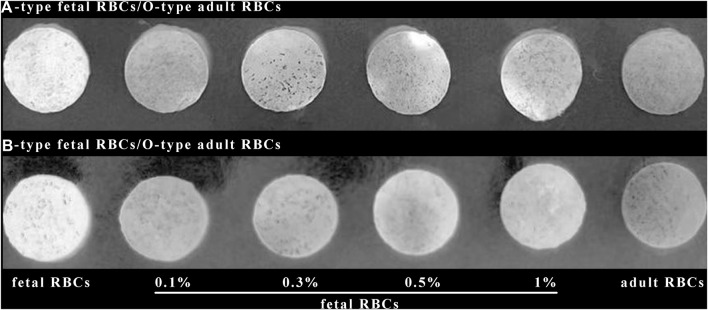
Using ImageJ to green channel the results of an ELISA based on a silk membrane.

**FIGURE 10:**
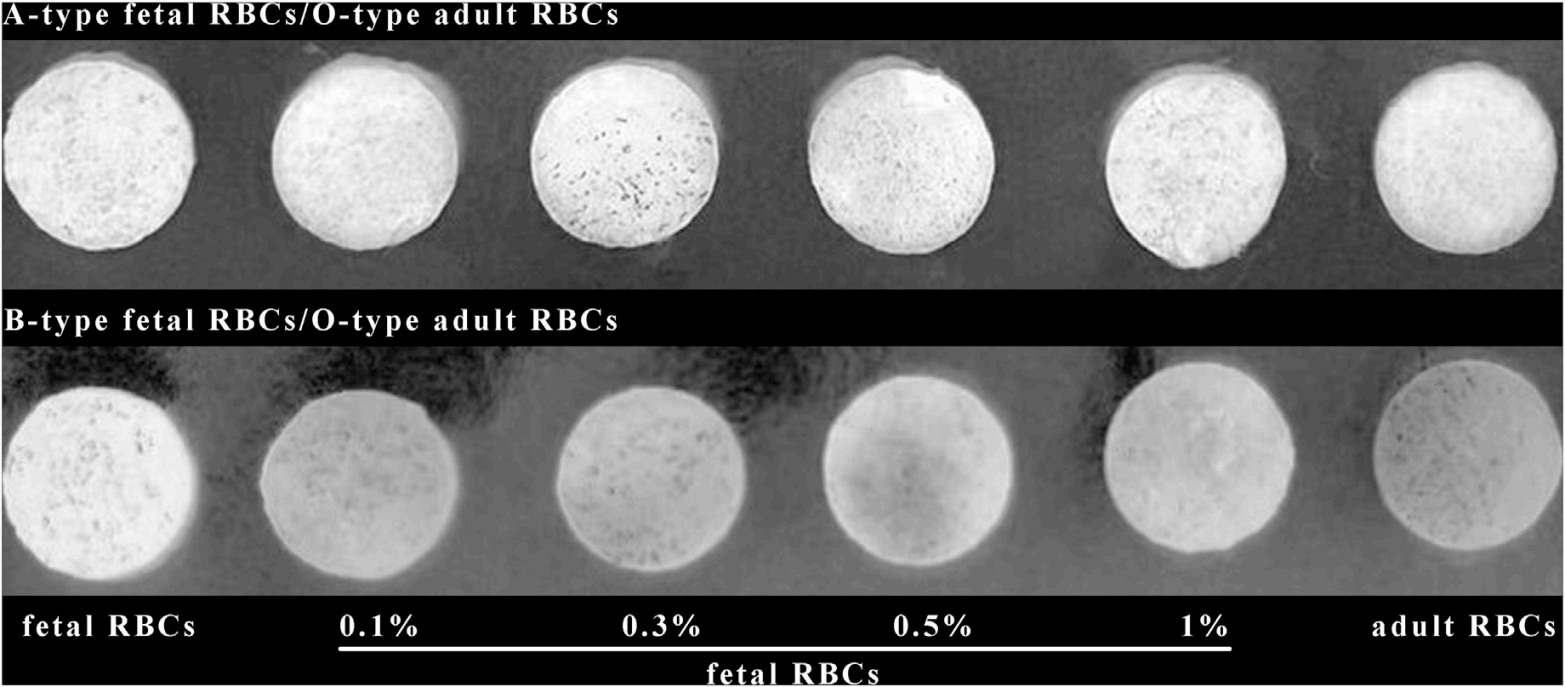
Using ImageJ to red channel the results of an ELISA based on a silk membrane.

**FIGURE 11:**
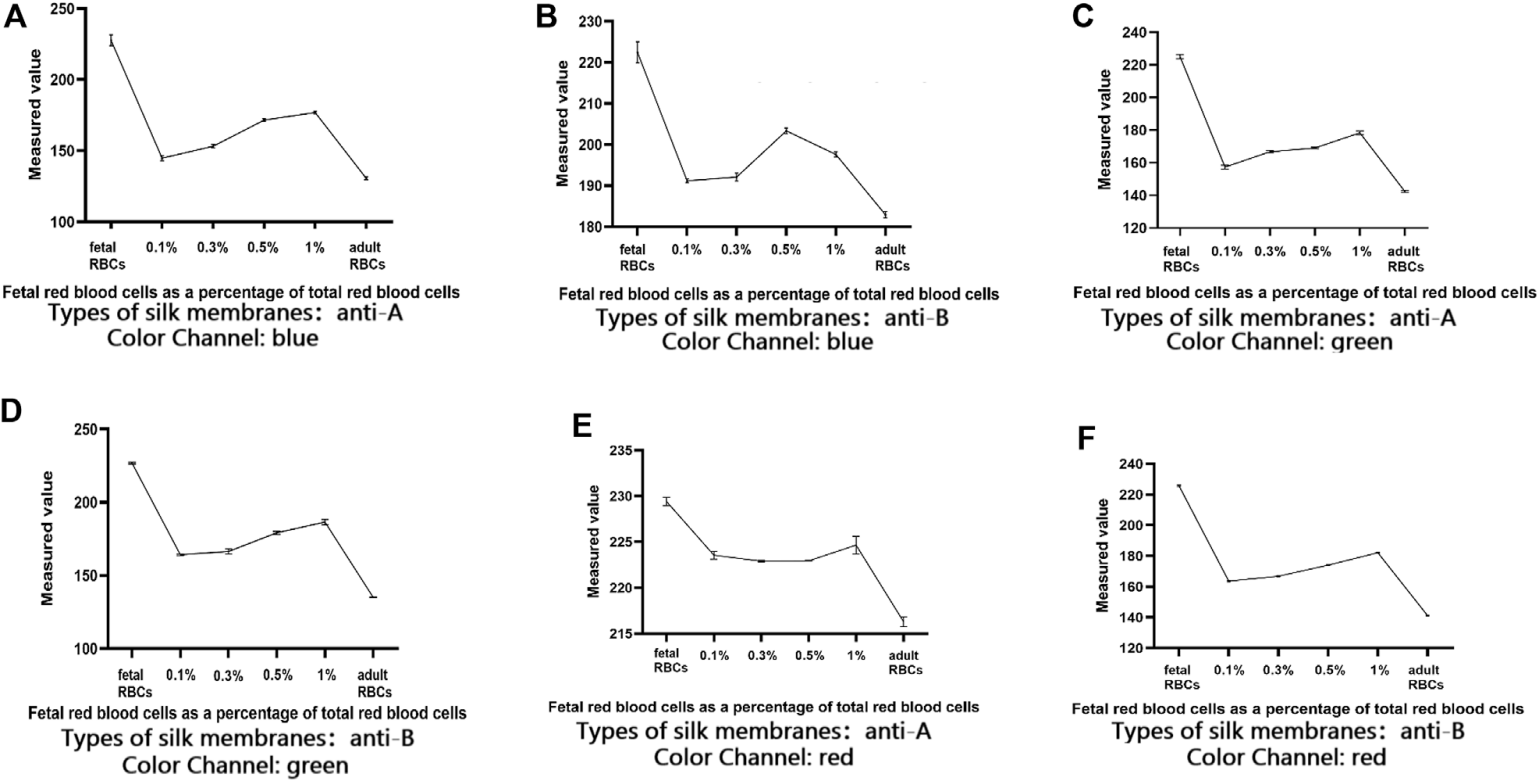
Quantification of FMH using an ELISA based on a silk membrane.

**FIGURE 12:**
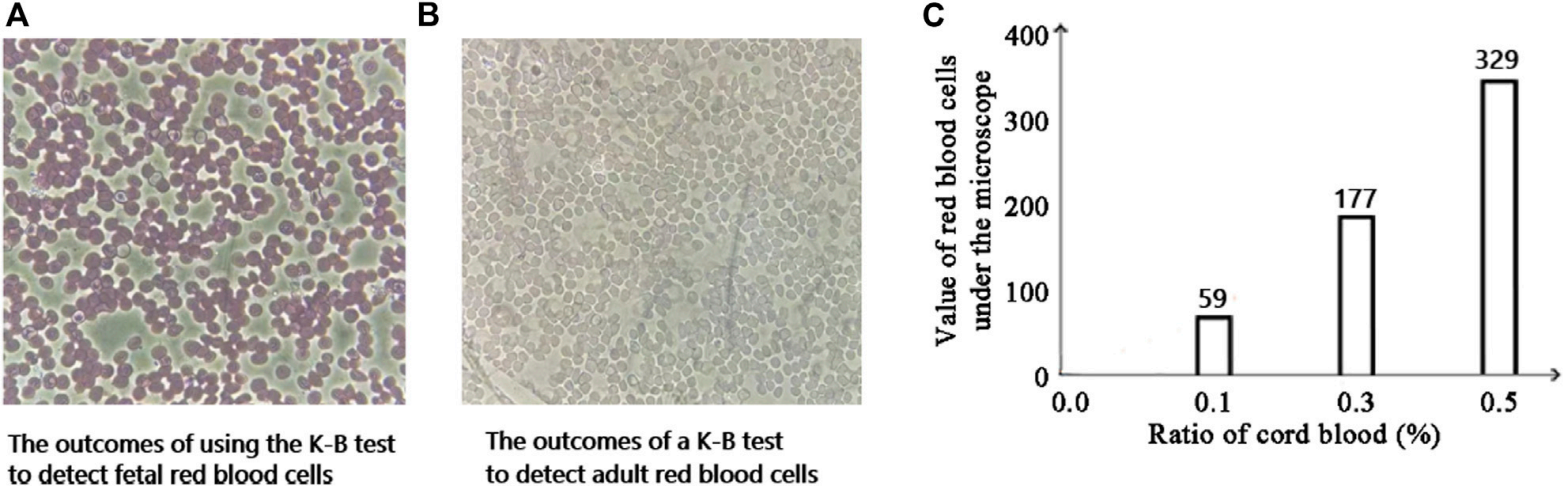
The results of FMH detected by K-B test.

**FIGURE 13:**
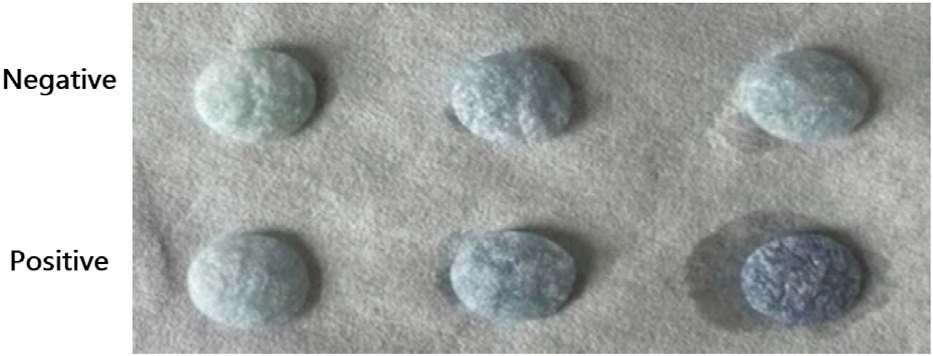
The negative and positive control results of an ELISA with a white silk membrane as basement membrane.

Figure 7 demonstrates that when the positive control and the negative control are established, the color changes of 0.1% fetal red blood cells and 0.3% fetal red blood cells are comparable, as are the color changes of 0.5% fetal red blood cells and 1% fetal red blood cells. Nonetheless, 0.5%–1% of fetal red blood cell test results are darker than 0.1%–0.3% of fetal red blood cell test results, and the range of FMH can be determined based on color. Moreover, each experiment was conducted four times with consistent results. Figure 11 illustrated the error bar. Additionally, this experiment quantified the experimental outcomes in greater detail. Using ImageJ software, the silk film was separated into “blue, green, and red” color channels and then inverted (see Figures 8–10). Then, the brightness data of inverted images in three distinct color channels was measured individually. GraphPad Prism 9 was used to manage the data (Figure 11). The optimal outcomes are as follows: the value of silk membrane treated with fetal red blood cells > the value of silk membrane treated with 1% fetal RBCs > the value of silk membrane treated with 0.5% fetal RBCs > the value of silk membrane treated with 0.3% fetal RBCs > the value of silk membrane treated with 0.1% fetal RBCs > the value of silk membrane treated with adult red blood cells. Compared to Figure 11, the silk film treated with anti-A produces superior results in the blue color channel (Figure 11A), while the silk film treated with anti-B produces superior results in the green color channel (Figure 11D). Consequently, ELISA based on a silk membrane can quantify with high sensitivity, high repeatability, and high dependability. Figure 12 shows the results of K-B test to detect different proportions of FMH. 12(A) is the result chart (positive control) of detecting fetal red blood cells by K-B test, which shows that the result of detecting fetal red blood cells by K-B test is red under the microscope; 12(B) is the result chart (negative control) of adult red blood cells detected by K-B test, which shows that adult red blood cells detected by K-B test are ghostly under the microscope. 12(C) is the result of detecting different proportions of fetal red blood cells in mixed blood by K-B test. Comparing Figure 7 with Figure 12, both K-B test and enzyme-linked immunosorbent assay based on silk membrane can detect 0.1% of fetal red blood cells, which can meet the needs of clinical detection of FMH sensitivity. In addition, ELISA based on silk membrane can be quantified by software, which was simple and quick, whereas the K-B experiment was subjective and requires the detection of 10,000 cells, which was time-consuming. This experiment replaced the blue silk film and yellow silk film used in the new method with white silk film and conducted parallel experiments to determine whether the color of the silk film affected the experimental outcomes. It was discovered that the silk film was colored similarly in both the positive and negative results. The reason for this is that haemoglobin was a type of pigment that can combine with silk membrane, altering the results of the experiment. This colored silk membrane eliminated the interference of haemoglobin and other pigments. Figure 13 displayed the outcomes.

### 3.6 A summary of the detection of maternal and fetal blood types in FMH using an ELISA based on silk membrane

Figure 7 demonstrates that ELISA based on a silk membrane developed in this experiment can detect the situation in which the fetal blood type is A or B and the maternal blood type is O. By using the anti-A/anti-B antibody attached to the silk membrane in this experiment, we can identify all instances in which the fetus has a different blood type than the mother. This experiment developed a novel method for prenatal detection of FMH, but it cannot determine the fetus’ blood type. Fortunately, the blood type of the fetus can be predicted based on the blood types of the father and mother, and the observation of the results is straightforward, so the experimental procedure will not be complicated. Table 1 displays the fetal blood types for which the father’s and mother’s blood types are known, as well as the test results for different blood types using new technology. When the mother’s blood type is AB or the same as that of the fetus, it is not necessary to test for fetal bleeding. When the mother’s blood type is A, a positive test result for 0.1% fetal bleeding of anti-B silk membrane has clinical significance; when the mother’s blood type is B, a positive test result for 0.1% fetal bleeding of anti-A silk membrane has clinical significance.

**TABLE 1:** Summary of the types of FMH detected by ELISA based on silk membrane (Silk membrane can be separated into anti-A and anti-B subtypes based on the conjugated antibody; +: positive result; -: negative result).

Maternal blood group	Fetal blood group	Test results of novel technology	
		Anti-A membrane silk	Anti-B membrane silk
O	A	+	-
O	B	-	-
O	AB	+	+
O	O	-	-
A	A	+	-
A	B	+	+
A	AB	+	+
A	O	+	-
B	A	+	+
B	B	-	+
B	AB	+	+
B	O	-	+

## 4 Discussion

In this experiment, the chemically treated silk membrane was combined with the cascade amplification enzyme-linked immunosorbent assay, and a new prenatal diagnosis method for FMH was developed, which was simple, fast, reliable, low-cost, easy to obtain, did not harm the health of the fetus or mother, and was anticipated to be applied to routine clinical detection. Silk membrane, as the matrix material of ELISA, not only capitalized on its excellent performance, but also took advantage of its pores’ ability to permeate red blood cells. In addition, the multilayer pore structure of natural silk membrane provided ample binding space for antigen and antibody. The experimental outcomes were in agreement with Wang’s article (Wanga et al., 2020). In contrast, the NC membrane on the market is incapable of penetrating red blood cells when compared to the treated silk membrane. Consequently, grinding natural silk film into paper resembling NC film facilitates the detection of FMH in future experiments. In addition, vacuuming degums the aperture and makes it more transparent. Anti-A and anti-B antibodies that bind specifically to fetal red blood cells in an ELISA utilizing a silk membrane are IgM blood group antibodies. The antibody can directly agglutinate with blood group antigen to form an immune complex that is bound to the pore diameter of the silk membrane. If anti-D antibody is attached to a silk membrane to combine with D antigen or if anti-fetal hemoglobin antibody is used to capture fetal hemoglobin, it must be linked to anti-human globulin or enzyme because anti-D antibody and anti-fetal hemoglobin antibody are IgG antibodies (Lemaitre et al., 2020; Ayenew, 2021).

This experiment has some limitations. This experiment merely has demonstrated that ELISA based on a silk membrane can detect whether the fetus’ blood type is A or B and the mother’s blood type is O. By using the anti-A/anti-B antibody attached to the silk membrane in this experiment, it is possible to identify all instances in which the fetus has a different blood type than the mother. The theoretical test results for various maternal and fetal blood types are presented in Table 1. Moreover, the clinical samples of FMH were not used for the experiment, mainly because FMH is a rare disease. However, in this experiment, different proportions of fetal red blood cells and adult red blood cells are used to simulate the real state of FMH, and the new enzyme-linked immunosorbant assay (ELISA) based on a silk membrane established in this experiment can detect 0.1% of FMH, which has the same sensitivity as K-B test, and is suitable for clinical sensitivity analysis of FMH detection.

In the experiment, the cross-sections were prepared by shearing off, which can be by immersed in liquid nitrogen, and later mannualy breaking. In future, the silk membrane in this study may be replaced by electrospun fibrous membrane, which can be more sensitive due to the smaller diameters of nanofibers, large surface and big porosity (Ge et al., 2023; Yu et al., 2023). This experiment replicates Wang’s idea of distinguishing anti -A antibody, anti -B antibody, and anti -D antibody by using different colored membranes, and uses different colored silk membranes to distinguish before detection in order to prevent detection errors caused by the inability to differentiate silk membranes treated with anti -A or anti -B antibodies. For instance, the silk membrane treated with anti-A antibody is blue and is used to detect erythrocyte antigen of type A; the silk membrane treated with anti-B antibody is yellow and is used to detect red blood cell antigen of type B. Obviously, it will be studied further in the future to use white silk films instead of different colors for experiments, so as to reduce color interference with test results.

## 5 Conclusion

This experiment resulted in the development of new enzyme-linked immunosorbent assay (ELISA) based on a silk membrane that is simple, rapid, reliable, sensitive, inexpensive, and requires no complex equipment. It can visually develop color based on a silk membrane and can differentiate fetal red blood cells from maternal red blood cells prior to delivery, allowing it to be used for prenatal detection of FMH.

## Data Availability

The original contributions presented in the study are included in the article/supplementary material, further inquiries can be directed to the corresponding author.
